# Effectiveness and safety of Levofloxacin containing regimen in the treatment of Isoniazid mono-resistant pulmonary Tuberculosis: a systematic review

**DOI:** 10.3389/fmed.2023.1085010

**Published:** 2023-06-20

**Authors:** Leeberk Raja Inbaraj, Hemant Deepak Shewade, Jefferson Daniel, Vignes Anand Srinivasalu, Jabez Paul, S. Satish, Richard Kirubakaran, Chandrasekaran Padmapriyadarsini

**Affiliations:** ^1^Department of Clinical Research, Indian Council of Medical Research-National Institute for Research in Tuberculosis, Chennai, India; ^2^Division of Health System Research, Indian Council of Medical Research – National Institute of Epidemiology, Chennai, India; ^3^Department of Pulmonary Medicine, Christian Medical College, Vellore, India; ^4^Prof. BV Moses Centre for Evidence Informed Healthcare and Health Policy, Christian Medical College, Vellore, India; ^5^Center for Biostastic and Evidence Based Medicine, Vellore, India

**Keywords:** fluoroquinolones, MDR-TB, resistant pulmonary Tuberculosis, Isoniazid resistance, levofloxacin

## Abstract

**Background:**

We aimed to determine the effectiveness and safety of the Levofloxacin-containing regimen that the World Health Organization is currently recommending for the treatment of Isoniazid mono-resistant pulmonary Tuberculosis.

**Methods:**

Our eligible criteria for the studies to be included were; randomized controlled trials or cohort studies that focused on adults with Isoniazid mono-resistant tuberculosis (HrTB) and treated with a Levofloxacin-containing regimen along with first-line anti-tubercular drugs; they should have had a control group treated with first-line without Levofloxacin; should have reported treatment success rate, mortality, recurrence, progression to multidrug-resistant Tuberculosis. We performed the search in MEDLINE, EMBASE, Epistemonikos, Google Scholar, and Clinical trials registry. Two authors independently screened the titles/abstracts and full texts that were retained after the initial screening, and a third author resolved disagreements.

**Results:**

Our search found 4,813 records after excluding duplicates. We excluded 4,768 records after screening the titles and abstracts, retaining 44 records. Subsequently, 36 articles were excluded after the full-text screening, and eight appeared to have partially fulfilled the inclusion criteria. We contacted the respective authors, and none responded positively. Hence, no articles were included in the meta-analysis.

**Conclusion:**

We found no “quality” evidence currently on the effectiveness and safety of Levofloxacin in treating HrTB.

**Systematic review registration:**

https://www.crd.york.ac.uk/prospero/display_record.php?ID=CRD42022290333, identifier: CRD42022290333.

## Introduction

Tuberculosis (TB), one of the important public health problems worldwide, affected 10 million people and killed 1.5 million individuals across the globe in 2020 (1). Drug-resistant Tuberculosis (DR-TB) is a major challenge for TB control and elimination. Multidrug resistance/ Rifampicin resistance (MDR/RR-TB) was found in 3–4% of new TB patients and 18–21% of previously treated cases in 2020, according to the World Health Organization (WHO) (1). WHO estimated that between 1995 and 2013, 9.5% of TB cases globally had Isoniazid resistance without Rifampicin resistance. The global average of Isoniazid resistance was 8.1% among newly diagnosed and 14% among previously treated patients (2). Isoniazid mono-resistance was found in 12% of pediatric cases globally, accounting for 120,000 new cases annually, reflecting the percentage observed among new adult cases (3). Unfortunately, Isoniazid mono-resistant TB (HrTB), a widely prevalent DR-TB, has not drawn similar attention as MDR TB in TB research and control strategies.

India's national TB report (2022) showed a cure rate and success rate of 55 and 83%, respectively, in patients with H-mono/poly resistance TB (4). Studies across the globe have reported outcome rates of 7–44% among these patients treated with first-line drugs. Isoniazid resistance is not only a risk for poor treatment outcomes but also predisposes to MDR-TB and polydrug resistance (5). A recent meta-analysis has shown that Isoniazid resistance reduced the probability of treatment success and increased the risk of relapse and progression to MDR-TB. Acquired drug resistance was 5.1 times (95% CI 2.3–11.0) higher among patients with Isoniazid resistance than patients with drug-susceptible Tuberculosis (6, 7).

In 2019, the WHO issued a conditional recommendation for a 6-month combination of Rifampicin, Ethambutol, Pyrazinamide, and Levofloxacin to treat patients with HrTB, based on data from 15 trials with a limited sample size. According to WHO, adding fluoroquinolones to a standard treatment regimen with or without Isoniazid improved treatment success while having no significant effect on mortality or acquired drug resistance (8). Subsequently, in 2019, Stagg et al. did a retrospective study and found no significant difference in adverse outcomes among HrTB patients treated with or without fluoroquinolones (9). Another school of thought suggests that fluoroquinolone is not required if HrTB patients are given a longer duration of treatment of 12 months (10). The argument against adding fluoroquinolone is based on the anticipated risk of introducing additional drug resistance when HrTB progresses into MDR- TB. It is also important to note that Rifampicin resistance was initially missed in 7.6% of HrTB patients (11).

Current evidence lacks clarity on the treatment regimen for HrTB. There is also uncertainty about the effectiveness of Levofloxacin on the treatment outcomes. We conducted this systematic review to determine the effectiveness of Levofloxacin containing first-line anti-tubercular drugs (ATT) in treating Isoniazid mono-resistance pulmonary TB.

## Methods

### Protocol and registration

We designed a systematic review and meta-analysis per preferred Reporting Items for Systematic Reviews and Meta-analyses (PRISMA) guidelines. We registered our protocol with the International Prospective Register of Systematic Reviews (PROSPERO) (CRD42022290333) (12, 13).

### Inclusion criteria

The eligible criteria were designed using PICO (participants, intervention, comparator and outcome) and included randomized control trials (RCTs) and cohort studies with exposed (received levofloxacin) and unxposed group (without levofloxacin). We included studies published in any language and from any country. We excluded case reviews, ecological studies, case-control, cross-sectional and other study designs. We focused on the studies that included adults (≥15 years) with HrTB on daily or intermittent anti-TB regimens with or without comorbid illnesses, either managed as in-patient or outpatient (P). The patients included in the studies must have been treated with Levofloxacin and a combination of the first-line ATT drugs (Rifampicin, Ethambutol, Pyrazinamide, Isoniazid), excluding the injectable drug streptomycin (I). The studies must have had a control group, or unexposed group in the case of cohort studies. They should have been treated with any combination of the first-line ATT drugs (Rifampicin, Ethambutol, Pyrazinamide, Isoniazid) but without Levofloxacin (C). Our outcomes of interest were; treatment success rate at the end of the treatment, mortality, recurrence, progression to MDR-TB and additional drug resistance during or after the treatment, and adverse outcomes (O).

### Data source and search strategy

We performed the search in MEDLINE (via PUBMED), EMBASE, Epistemonikos, Google Scholar, Clinical trials registry, and Cochrane Central Registrar of Controlled Trials (CENTRAL) in Cochrane library from January 1, 1990, to September 2021. We did not include studies published before 1990 as Levofloxacin was not used for TB treatment earlier. The search strategies (Supplementary material 1) were developed based on our PICO, and information specialists did the literature search. We also manually searched the reference list of the selected articles for additional studies missed during the initial electronic search. The bibliographies of all full-text articles and previous systematic reviews [Stagg et al. (14), Georgia et al. (15), and Fregonese et al. (16)] on HrTB outcomes were also examined for potential articles.

### Data collection

#### Study selection

**Figure 1:**
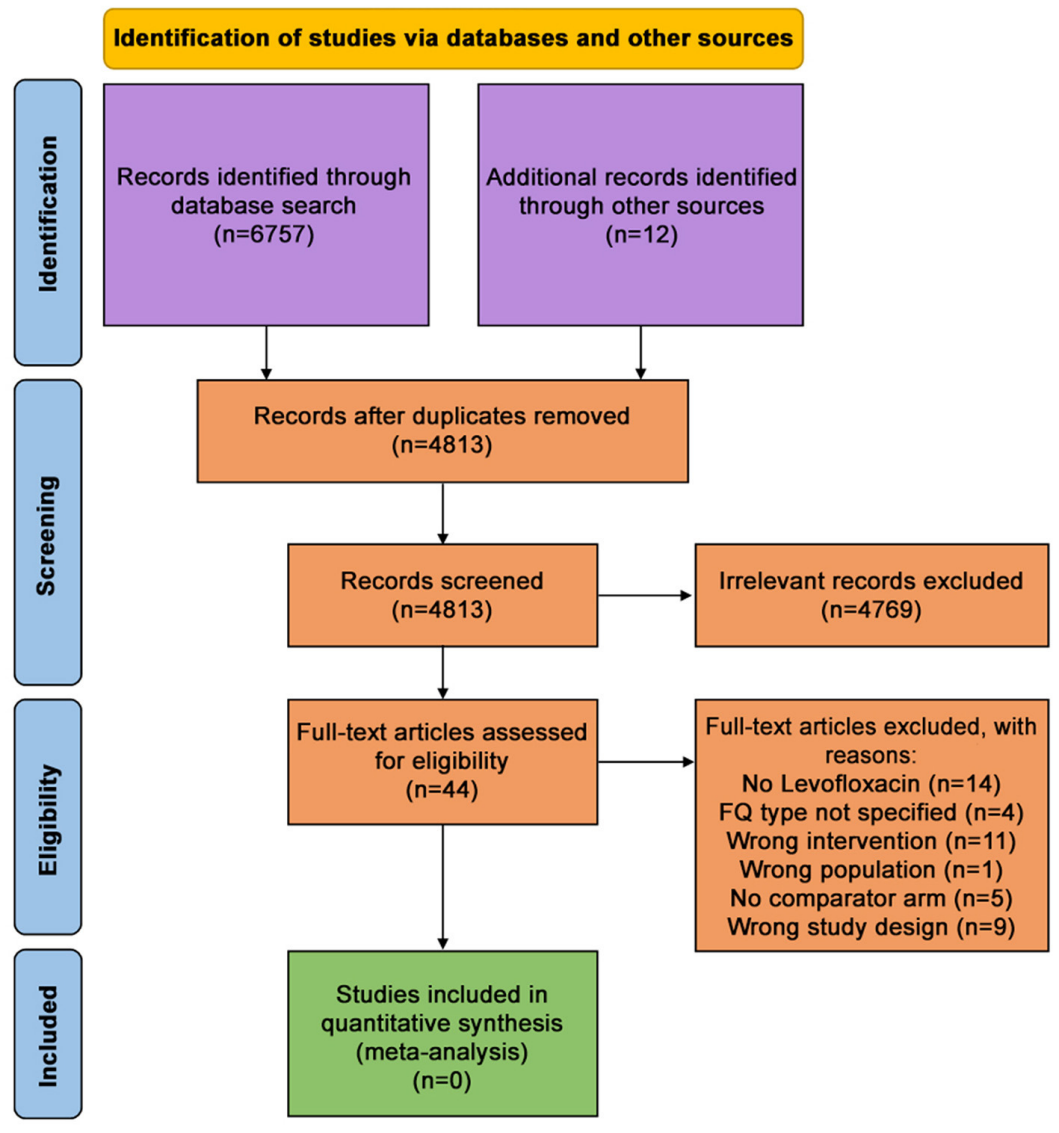
PRISMA flow diagram.

Titles/abstracts provided by the search experts (JP/SS) were imported to the Rayyan software, and duplicates were excluded. Two independent reviewers (JD/VA) screened the titles and abstracts using our PICO criteria and shortlisted potential publications for detailed assessment. Two reviewers (JD/VA) further analyzed the shortlisted articles independently and documented specific reasons for exclusion. Discrepancies were resolved along with a third investigator (LR). All decisions made during the selection process were recorded and presented in a PRISMA flow diagram (Figure 1).

We reviewed the primary data in the supplementary available, and if not available, we sent requests for primary data. We sent three additional reminder e-mails once a fortnight and waited for a reply from the authors for a maximum of 45 days after the first e-mail.

#### Data extraction

Two of our independent reviewers (JD/VA) planned to extract the data from the included studies into a data extraction form (Supplement material 2). We also proposed to have a third reviewer (LR) to resolve the discrepancies.

#### Risk of bias assessment

The plan was to assess publication bias by plotting effect estimates from included studies on a funnel plot and will utilize Begg's or Egger's Test (17, 18). We planned to assess cohort studies using the Newcastle-Ottawa Scale and Cochrane risk of bias tool 2.0 for RCTs (19, 20).

The studies were planned to categorize into three groups depending on the level of bias: low, medium or high risk of bias. We proposed assessing the quality of the evidence using the Grades of Recommendation, Assessment, Development and Evaluation (GRADE) methodology by two independent reviewers (21). However, no articles were included, and the risk of bias assessment was not done.

### Statistical analysis

We aimed to perform analyses according to the recommendations of the Cochrane Handbook for Systematic Reviews of Interventions using Review Manager 5.4 (RevMan5.4) software (22). We intended to record the mean, standard deviation and total participants for continuous outcomes such as “cured' and treatment completed” in both treatment and control groups and perform analyses using standardized mean difference. We planned to record the number of events and total participants for dichotomous outcomes such as mortality, relapse and toxicity and pool the data using a risk ratio (RR) with 95% CI.

We proposed to use the fixed-effect model for dichotomous data (Mantel-Haenszel method) and the inverse variance method for continuous data (23). The plan was to assess the heterogeneity of treatment effects between trials using the I^2^ statistic and visual examination to quantify the statistical heterogeneity. We also scheduled to do sub-group analysis for TB with HIV, TB with diabetes mellitus, newly diagnosed and previously treated TB, treated with an intermittent or daily regimen, and low or high phenotypic resistance. Data was not extracted because there were no included studies; hence statistical analysis was not performed.

### Operational definitions

Operational defintions were taken from WHO's Definitions and reporting frame work for TB (24).

***Treatment success:*** Sum of cure rate and treatment completion.

***Cure:*** “A pulmonary TB patient with bacteriologically confirmed TB at the beginning of treatment who was smear- or culture-negative in the last month of treatment and on at least one previous occasion.”

***Treatment completed:*** “A TB patient who completed treatment without evidence of failure, but with no record to show that sputum smear or culture results in the last month of treatment and on at least one previous occasion were negative, either because tests were not done or because results are unavailable.”

***Treatment failure:*** “A patient who is sputum culture positive at 5 months or later during treatment.”

***Died:*** “A TB patient who dies for any reason before starting or during the course of treatment.”

***Default/ Loss to follow-up:*** “A patient who did not start treatment or whose treatment was interrupted for two consecutive months or more.”

***Not evaluated:*** “A patient for whom no treatment outcome is assigned. This includes cases ‘transferred out' to another treatment unit as well as cases for whom the treatment outcome is unknown to the reporting unit.”

***TB recurrence:*** Defined as “the presence of a new episode of TB disease in a TB patient who was declared cured or treatment completed and remained TB disease-free for a minimum of 6 months after the end of the most recent anti-TB treatment. This includes bacteriologically confirmed cases and clinically diagnosed cases.”

## Results

We found no RCT or cohort studies fitting our inclusion criteria. No ongoing trials or cohort studies are fulfilling our inclusion criteria. Our search in MEDLINE, EMBASE, Cochrane Database of systematic review, and Google Scholar yielded 6,757 records, and we collected 12 records from other sources. After removing duplicates, 4,813 records underwent titles and abstracts screen (Figure 1). We excluded 4,768 records and retained 44 records for full-text screening. Two reviewers screened these 44 records, and all of them were excluded. The reason for exclusion and study characteristics are described in Table 1. Though none of the records fully matched our inclusion criteria, eight articles partially fulfilled the inclusion criteria. For clarifications, we contacted the respective authors of those nine studies by e-mail and followed it with three reminders fortnightly. However, none responded positively, and hence we did not include them (Table 2).

**Table 1:** Characters of excluded study with reasons for exclusion.

**S. no**	**References, country**	**Study design**	**P**	**I**	**C**	**O**	**Comment**
1	Chien et al. (25), Taiwan	Retrospective cohort	Y	N	N	Y	•Multiple regimens were used. Type fluoroquinolone not specified. •No standard regimen followed. •Intervention did not match PICO. •Injectables were given for a few patients
2	WHO Treatment Guidelines (26), Switzerland	Guidelines	N	N	N	N	•Not a Primary research study •Wrong design
3	Sayfutdinov et al. (27), Uzbekistan	Retrospective cohort	Y	N	N	Y	•Intervention not matching PICO & Injectables used •No comparator arm
4	Schechter et al. (28), United States of America	retrospective cohort	N	N	N	Y	•Type of fluoroquinolone not specified, •Intervention did not match PICO. •Injectables were given for a few patients. •Some were wrong population – Extra pulmonary TB
5	Diel and Schluger (10), Germany	Review article	N	N	N	N	•Not a Primary Research study Wrong design
6	Stagg et al. (29), United Kingdom	Conference abstract	N	N	N	N	•Not a Primary Research study Wrong design
7	Migliori et al. (30), Italy	Review article	N	N	N	N	•Not a Primary Research study Wrong design
8	Gegia et al. (15), Switzerland	Review article	N	N	N	N	•Not a Primary Research study •Wrong design •Wrong intervention – no Levofloxacin •Multiple regimens were used
9	Wilson et al. (31), Australia	Retrospective case series	N	N	N	Y	•Type of fluoroquinolone not specified •Intervention did not match PICO. •Injectables were given for a few patients. •Some were wrong population – Extra pulmonary TB
10	Báez-Saldaña et al. (32), Mexico	Prospective cohort	Y	N	Y	Y	•Wrong intervention arm – no fluoroquinolone
11	Villegas et al. (33), Peru	Prospective cohort	Y	N	Y	Y	•Wrong intervention arm – Multiple regimens used
12	Tabarsi et al. (34), Iran	Retrospective cohort	Y	N	Y	Y	•Wrong intervention arm – no fluoroquinolone
13	Escalante et al. (35), United States of America	Retrospective cohort	Y	N	Y	Y	•Wrong intervention •Type of fluroquinolone is not specified
14	Swai et al. (36), Kenya	RCT	Y	N	N	Y	•Wrong intervention and comparison •No fluroquinolone
15	Nolan et al. (37), United States of America	Review article	N	N	N	N	•Not a Primary Research study Wrong design
16	Binkhamis et al. (38), Saudi Arabia	Retrospective cohort	Y	N	N	Y	•Wrong intervention and comparison •No fluroquinolone
17	van der Heijden et al. (39), South Africa	Retrospective cohort	Y	N	Y	Y	•Wrong intervention •No fluroquinolone
18	Hoopes et al. (40), United States of America	Retrospective cohort	Y	N	N	Y	•Wrong intervention •No fluroquinolone
19	Munang et al. (41), United Kingdom	Retrospective cohort	Y	N	Y	Y	•No fluoroquinolone in 6-month regime for comparison
20	Cattamanchi et al. (42), United States of America	Retrospective cohort	Y	N	Y	Y	•No fluoroquinolone for comparison
21	Fox et al. (43), Israel	Retrospective cohort	Y	N	N	Y	•Regimen not defined
22	Fregonese et al. (16), Canada	Review article	N	N	N	N	•Not a Primary Research study •Type of quinolone not specified
23	Reves et al. (44), United States of America	Prospective cohort	Y	N	N	Y	•No comparator arm
24	Kim et al. (45), South Korea	Retrospective cohort	Y	N	Y	Y	•No Levofloxacin in comparator arm •Multiple regimens used
25	Garcia-Prats et al. (46), South Africa	Prospective cohort	N	N	N	Y	•Wrong population – Pediatric •Multiple regimens
26	Nagu et al. (47), Tanzania	Retrospective cohort	Y	N	N	Y	•No Levofloxacin in comparator arm •Multiple regimens
27	Jhun and Koh (5), South Korea	Review article	N	N	N	N	•Not a Primary Research study
28	LoBue et al. (48), United States of America	Retrospective cohort	Y	N	N	Y	•No Levofloxacin in comparator arm •Multiple regimens used
29	Stagg et al. (9), United Kingdom	Retrospective cohort	Y	N	N	Y	•Rifamycin instead of Rifampicin •The duration of treatment is 12 months
30	Thai et al. (49), Vietnam	Retrospective cohort	Y	N	N	Y	•No Levofloxacin in comparator arm •Multiple regimens were used
31	Huyen et al. (50), Vietnam	Prospective cohort	Y	N	N	Y	•No comparator arm
32	Ormerod et al. (51), United Kingdom	Retrospective cohort	Y	N	Y	Y	•No Levofloxacin in comparator arm •Multiple regimens used
33	Bai et al. (52), Taiwan	Retrospective cohort	Y	N	N	Y	•No comparator arm
34	Salindri et al. (53), United States of America	Retrospective cohort	Y	N	N	Y	•No comparator arm
35	Lai et al. (54), Taiwan	Letter to the editor	N	N	N	N	•Wrong study design
36	Maguire et al. (55), United Kingdom	Case control study	Y	N	N	N	•Wrong study design
37	Cornejo Garcia et al. (56)^*^, Peru	Retrospective cohort	Y	N	N	Y	•Wrong intervention •Comparator arm also had Levofloxacin
38	Edwards et al. (57)^*^, Canada	Retrospective cohort	Y	N	N	Y	•Multiple combination of first line ATT used •Type of fluroquinolone not specified
39	Kim et al. (58)^*^, South Korea	Retrospective cohort	Y	N	N	Y	•Wrong intervention •Comparator arm was drug sensitive TB
40	Lee et al. (59)^*^, South Korea	Retrospective cohort	Y	N	N	Y	•Combination of first line drugs used •Both Levofloxacin and moxilfloxacin used
41	Romanowski et al. (60)^*^, Canada	Retrospective cohort	Y	N	N	Y	•Multiple combination of first line drugs used •Included both pulmonary and extrapulmonary TB
42	Bang et al. (61)^*^, Denmark	Retrospective cohort	Y	N	N	Y	•Patients with INH mono and poly resistance were included •Different drug regimens were used
43	Saito et al. (62)^*^, Japan	Retrospective cohort	N	N	N	Y	•Included patients with INH resistance and drug sensitive TB •Different drug regimens were used •No comparator arm with Levofloxacin
44	Bachir et al. (63)^*^, France	Retrospective case-control	N	N	N	Y	•Compared patients with INH resistance and drug sensitive TB •Included both pulmonary and extra pulmonary TB.

* Authors contacted for data.

**Table 2:** Summary of current evidence on the effectiveness of Levofloxacin in the treatment of HrTB.

**References**	**Country**	**No. of patients with HrTB**	**Regimen**	**Comparator regimen**	**Outcome**	**Remarks**
Cornejo Garcia et al. (56)	Peru	947	LfxREZ (791 patients)	LfxREZ with second-line injectable (156 patients)	The cure rate was similar in both groups. Mortality was lower in the group only with Levofloxacin.	Included patients with extrapulmonary TB and did not have a comparative arm without Levofloxacin
Lee et al. (59)	South Korea	140	FQ (75 patients)	Combination of first-line drugs	FQ group had a significant difference in the treatment response in terms of improvement in the chest, X-rays negative conversion in sputum AFB smears compared to those who did not receive FQ. The crude and adjusted proportion of unfavorable outcomes was lower for patients treated with FQs.	The patients with FQ regimens were on treatment for a longer duration, and several regimens were used, which limited the effective comparison between the groups.
Bang et al. (61)	Denmark	111	FQ (40 patients)	3RE(H)Z/3RE(Z) or FQ along with REZ	90% treatment success rate	
Romanowski et al. (60)	Canada	165	FQ (40 patients)	30 different regimens of first-line ATT	FQ-containing regimen had no relapse in their cohort	The variety of treatment regimens received by these patients makes it difficult to conclude the effectiveness of FQ containing regimen for the treatment of HrTB.
Edwards et al. (57)	Canada	168	FQ (69 patients)	Different combinations of first-line ATT	Compared to non-FQ containing regimen, no difference in unsuccessful treatment outcomes.	Both Moxifloxcacin or Levofloxacin were used. More than half used FQ intermittently during the continuation phase
Kwak et al. (3)	South Korea	195	FQ (53 patients)	Different combinations of first-line ATT	There were no significant differences in favorable outcomes between the patients treated with FQ and those who did not.	Patients were included from 2005 before the revised guidelines of WHO.

Lfx, Levofloxacin; R, Rifampicin; E, Ethambutol; Z, Pyrazinamide; FQ, Fluroquinolones; ATT, anti-tubercular therapy; HrTB, Isoniazid mono-resistant tuberculosis; WHO, World Health Organization.

### Risk of bias in included studies

No included studies.

### Effects of intervention

No data available.

## Discussion

All the records retained for full-text review were excluded, and we discussed a few studies that closely matched our PICO. Cornejo Garcia et al. did a retrospective analysis of HrTB patients in Peru from 2012 to 2014. Of 947 patients assigned treatment outcomes, 791 received Levofloxacin (Levofloxacin, Rifampicin, Ethambutol, and Pyrazinamide), and 156 received an injectable in addition to Levofloxacin (Levofloxacin, Rifampicin, Ethambutol, and Pyrazinamide plus second- line injectable). The cure proportion was almost similar in both groups (34.4 vs. 34.6%). However, the mortality was lower in the group only with Levofloxacin (0.8 vs. 7.1%), and additional use of second-line injectable with Levofloxacin was associated with higher odds of [Odd's ratio (OR): 0.46; 95% CI 0.31–0.70, *p* < 0.05] unfavorable outcomes. This study included even extra pulmonary HrTB and did not compare the effectiveness with first-line ATT without Levofloxacin (56).

When 75 of 140 patients with HrTB received fluoroquinolones (FQ), there was a significant difference in treatment response in terms of chest X-ray improvement (69.2 vs. 48 %, *p* 0.01) and negative conversion in sputum AFB smears (59.3 vs. 31.3%) compared to those who did not receive FQ. Patients treated with FQs had a decreased crude (8.5 vs. 15.4%, *p* 0.01) and adjusted proportion (1.5 vs. 7.4%, *p* 0.037) of unfavorable outcomes. However, in this retrospective analysis between 2005 and 2012, the patients with the FQ regimens were on treatment for a longer duration, and several different regimens were used, limiting the effective comparison between the two groups. FQ group had received either moxifloxacin or Levofloxacin exclusively. Moreover, Isoniazid was discontinued in 84.3% (118/140) patients after a median of 2.1 months, which could have contributed to favorable outcomes (59).

Another retrospective analysis from South Korea compared treatment regimens between patients with Isoniazid resistant and susceptible Tuberculosis and reported a significant difference in the reduction of unfavorable outcomes when the latter group was treated with continuing Pyrazinamide and/or adding a FQ. The former group had a more smear-positive rate and was treated by discontinuing Pyrazinamide with or without Ethambutol. However, the sample size was too small (86 TB patients), and these unfavorable outcomes were not bacteriologically confirmed, and it was impossible to validate the diagnosis as it was a retrospective analysis (58).

Thirty-six (90%) patients were found to have been treated successfully when 111 patients with mono and poly resistance to HrTB were analyzed retrospectively in Denmark. The most common regimen used was the modified standard HREZ (Isoniazid (H), Rifampicin (R), Ethambutol (E), Pyrazinamide (Z)) given for 6 months as 3RE(H)Z/3RE(Z) or FQ along with REZ (61). FQ (Levofloxacin, Moxifloxacin, or Gatifoxacin) containing regimen had no relapse compared to 30 different regimens without FQ when 165 patients with HrTB were analyzed in Canada. The variety of treatment regimens received in this cohort played a considerable limitation to draw conclusions on the effectiveness of the FQ-containing regimen for treating HrTB (60).

On the contrary, of 69 patients who were initiated on FQ containing regimen, there was no difference in unsuccessful treatment outcomes compared to non-FQ-containing regimens (5.8 vs. 13.8%, OR 0.4; 95% CI 0.1–2.3, *p*- 0.23). This analysis included 168 patients with pulmonary and extrapulmonary and those who received moxifloxacin and Levofloxacin in Canada. Moreover, FQ was used intermittently during the continuation phase (57).

Similarly, Kawak et al. did not find significant differences in favorable outcomes between FQ group and the non-FQ group in South Korea when they analyzed the outcomes of 195 patients with HrTB. FQ was probably administered (36.3%) to patients with extensive disease or severe adverse reactions as the patients were included from 2005 before WHO's revised guidelines. Additionally, as in the studies mentioned above the sample size was too small, so the association between treatment and the outcomes was limited (64). In a recent study of 626 HrTB patients, Stagg et al. found no significant difference in the odds of conversion to negative sputum AFB smears between the two groups (cluster-specific OR 1.02; 95% CI 0.59–1.77; *p*-0.93). The authors reported on the outcomes of 594 patients for whom regimen information was available, 330 of whom were treated with (H)RfZE (Rf- rifamycins) and 211 with (H)RfZE and FQ (Moxifloxacin) (9).

Though the intervention of our interest was Levofloxacin, most of these retrospective analyses had a mix of patients with pulmonary and extrapulmonary TB who received different fluoroquinolones (Levofloxacin, moxifloxacin or gatifloxacin). It is likely that there are not enough observational studies and RCTs as the recommendation of the WHO to include Levofloxacin only in 2018 (8). The recommendation was based on individual participant data (IPD) meta-analysis of 5,418 patients from 33 global data sets. The WHO reported that when Z was given for >4 months, additional use of FQ was associated with higher odds of treatment success. The recommendations were with very low certainty of the evidence, and Levofloxacin was proposed as a first choice due to its safety profile and fewer known drug interactions compared to moxifloxacin. Similarly, there is no contraindication for Levofloxacin when used with other antiretroviral drugs, unlike moxifloxacin.

Fluoroquinolones, particularly Levofloxacin, have played an essential role in treating drug-resistant Tuberculosis, such as HrTB and MDR-TB. WHO guidelines based on individual participant data (IPD) meta-analysis and a few observational studies have shown better outcomes with levofloxacin in HrTB. However, currently, there is no sufficient evidence on the safety and efficacy of Levofloxacin in treating HrTB. Since the question of effectiveness and safety could be answered precisely through RCTs, we hope robust RCTs are planned in the future, and we will be able to generate evidence for the practice in the future. We found a good number of retrospective observational studies. However, we could not perform a meta-analysis since neither the comparator arm nor the intervention were of our interest.

Our systematic review had robust methodology, however had a few limitations. We extracted articles from four databases, and few more additional data bases could have yielded more articles. We had a strict inclusion and exclusion criteria, probably one of the reasons that we did not find any articles that were suitable to be included in the review.

## Conclusion

Our review calls for well-designed randomized control trials and robust prospective pragmatic studies to determine the effectiveness of the use of Levofloxacin in HrTB and long-term follow up studies to evaluate the treatment success and TB recurrence.

## Data availability statement

The original contributions presented in the study are included in the article/Supplementary material, further inquiries can be directed to the corresponding authors.

## Author contributions

LI, CP, and HS conceived the study. LI wrote the protocol, developed the data extraction form, played the role of arbitrator in the screening of the article, coordinated the review, and prepared the preliminary draft of the manuscript. HS reviewed the protocol and data collection tool and trained the reviewers. JD and VS were involved in the screening of the article and reviewed the draft of the manuscript. JP and SS developed the search strategy and extracted the articles. RK reviewed the protocol and wrote the statistical analysis and functioned as a methodological expert. CP supervised the project and reviewed the protocol and manuscript. All authors revised the work for important intellectual content and agreed to be accountable for all aspects of the work. All authors read and approved the final manuscript.

## References

[1] World Health Organization. Global Tuberculosis Report 2021. World Health Organization (2021). Available online at: https://apps.who.int/iris/handle/10665/346387 (accessed October 20, 2022).

[2] World Health Organization. Global Tuberculosis Report 2014. World Health Organization (2014). Available online at: http://apps.who.int/iris/handle/10665/137094 (accessed October 20, 2022).

[3] Yuen CM Jenkins HE Rodriguez CA Keshavjee S Becerra MC. Global and regional burden of isoniazid-resistant tuberculosis. Pediatrics. (2015) 136:e50–9. 10.1542/peds.2015-0172 26034243 PMC4485010

[4] Ministry of Health Family Welfare Government of India Coming Together to End TB Altogether. (2022). Available online at: https://tbcindia.gov.in/WriteReadData/IndiaTBReport2022/TBAnnaulReport2022.pdf (accessed October 20, 2022).

[5] Jhun BW Koh W-J. Treatment of isoniazid-resistant pulmonary tuberculosis. Tuberc Respir Dis. (2020) 83:20. 10.4046/trd.2019.0065 31905429 PMC6953491

[6] Menzies D Benedetti A Paydar A Martin I Royce S Pai M . Effect of duration and intermittency of rifampin on Tuberculosis treatment outcomes: a systematic review and meta-analysis. PLoS Med. (2009) 6:e1000146. 10.1371/journal.pmed.1000146 19753109 PMC2736385

[7] Menzies D Benedetti A Paydar A Royce S Pai M Burman W . Standardized treatment of active tuberculosis in patients with previous treatment and/or with mono-resistance to isoniazid: a systematic review and meta-analysis. PLoS Med. (2009) 6:e1000150. 10.1371/journal.pmed.1000150 20101802 PMC2736403

[8] World Health Organization. WHO Consolidated Guidelines on Drug-Resistant Tuberculosis Treatment. World Health Organization (2019). Available online at: https://apps.who.int/iris/handle/10665/311389 (accessed October 20, 2022).

[9] Stagg HR Bothamley GH Davidson JA Kunst H Lalor MK Lipman MC . Fluoroquinolones and Isoniazid-resistant Tuberculosis: implications for the 2018 WHO guidance. Eur Respir J. (2019) 54:1900982. 10.1183/13993003.00982-2019 31371444 PMC6785706

[10] Diel R Schluger NW. Is adding fluoroquinolones to regimens for treating Isoniazid-resistant Tuberculosis necessary? Eur Respir J. (2019) 54:1901494. 10.1183/13993003.01494-2019 31601721

[11] Van Deun A Decroo T Kya Jai Maug A Hossain MA Gumusboga M Mulders W . The perceived impact of Isoniazid resistance on outcome of first-line Rifampicin-throughout regimens is largely due to missed Rifampicin resistance. PLoS ONE. (2020) 15:e0233500. 10.1371/journal.pone.0233500 32421749 PMC7233532

[12] Moher D Shamseer L Clarke M Ghersi D Liberati A Petticrew M . Preferred reporting items for systematic review and meta-analysis protocols (PRISMA-P) 2015 statement. Syst Rev. (2015) 4:1. 10.1186/2046-4053-4-1 25554246 PMC4320440

[13] Effectiveness of Levofloxacin (LFX) Containing Drug Regimens in the Treatment of Isoniazid (INH) Mono Resistance Pulmonary Tuberculosis (Hr-TB): A Systematic Review Meta-Analysis. (2022). Available online at: https://www.crd.york.ac.uk/prospero/display_record.php?ID=CRD42022290333 (accessed October 20, 2022).

[14] Stagg HR Harris RJ Hatherell H-A Obach D Zhao H Tsuchiya N . What are the most efficacious treatment regimens for Isoniazid-resistant Tuberculosis? A systematic review and network meta-analysis. Thorax. (2016) 71:940–9. 10.1136/thoraxjnl-2015-208262 27298314 PMC5036252

[15] Gegia M Winters N Benedetti A van Soolingen D Menzies D. Treatment of Isoniazid-resistant Tuberculosis with first-line drugs: a systematic review and meta-analysis. Lancet Infect Dis. (2017) 17:223–34. 10.1016/S1473-3099(16)30407-8 27865891

[16] Fregonese F Ahuja SD Akkerman OW Arakaki-Sanchez D Ayakaka I Baghaei P . Comparison of different treatments for Isoniazid-resistant Tuberculosis: an individual patient data meta-analysis. Lancet Respir Med. (2018) 6:265–75. 10.1016/S2213-2600(18)30078-X 29595509 PMC9017096

[17] Begg CB Mazumdar M. Operating characteristics of a rank correlation test for publication bias. Biometrics. (1994) 50:1088–101. 10.2307/2533446 7786990

[18] Egger M Smith GD Schneider M Minder C. Bias in meta-analysis detected by a simple, graphical test. BMJ. (1997) 315:629–34. 10.1136/bmj.315.7109.629 9310563 PMC2127453

[19] Wells G Wells G Shea B Shea B O'Connell D Peterson J. The Newcastle-Ottawa Scale (NOS) for Assessing the Quality of Nonrandomized Studies in Meta-Analyses. (2014). Available online at: https://www.ohri.ca/programs/clinical_epidemiology/oxford.asp (accessed October 20, 2022).

[20] Sterne JAC Savović J Page MJ Elbers RG Blencowe NS Boutron I . RoB 2: a revised tool for assessing risk of bias in randomized trials. BMJ. (2019) 366:l4898. 10.1136/bmj.l4898 31462531

[21] GRADE Working Group,. The Grading of Recommendations Assessment, Development Evaluation (short GRADE). (2016). Available online at: https://www.gradeworkinggroup.org/ (accessed October 20, 2022).

[22] The Cochrane Collaboration Review Manager Web (RevMan Web). (2020). Available online at: https://training.cochrane.org/online-learning/core-software/revman (accessed October 20, 2022).

[23] Deeks JJ Higgins JPT Altman DG. Chapter 10: analyzing data and undertaking meta-analyses. In:Higgins JPT Thomas J Chandler J Cumpston M Li T Page MJ Welch VA, editors. Cochrane Handbook for Systematic Reviews of Interventions version 6.2. Cochrane. (2021). Available online at: www.training.cochrane.org/handbook (accessed February 2021).

[24] Definitions and Reporting Frame Work for TB – Revised 2013. World Health Organization. Available online at: https://apps.who.int/iris/bitstream/handle/10665/79199/9789241505345_eng.pdf?sequence=1&isAllowed=y (accessed November 08, 2021).

[25] Chien JY Chen YT Wu SG Lee JJ Wang JY Yu CJ. Treatment outcome of patients with isoniazid mono-resistant tuberculosis. Clin Microbiol Infect. (2015) 21:59–68. 25636929 10.1016/j.cmi.2014.08.008

[26] World Health Organization. WHO Treatment Guidelines for Isoniazid-Resistant Tuberculosis: Supplement to the WHO Treatment Guidelines for Drug-Resistant Tuberculosis. World Health Organization (2018). Available online at: https://www.who.int/publications/i/item/9789241550079 30285343

[27] Sayfutdinov Z Kumar A Nabirova D Gadoev J Turaev L Sultanov S . Treatment Outcomes of Isoniazid-Resistant (Rifampicin Susceptible) Tuberculosis Patients in Uzbekistan, 2017–2018. Int J Environ Res Public Health. (2021) 18:2965. 33799350 10.3390/ijerph18062965 PMC8001662

[28] Schechter MC Bizune DJ Kagei M Machaidze M Holland DM Oladele A . Time to sputum culture conversion and treatment outcomes among patients with isoniazid-resistant tuberculosis in Atlanta, Georgia. Clin Infect Dis. (2017) 65:1862–71. 29020173 10.1093/cid/cix686 PMC5850645

[29] Stagg H Abbara A Alexander E Baker L Booth H Bothamley GH . S152 Investigating the role of fluoroquinolones in the treatment of isoniazid resistant tuberculosis: implications for the 2018 WHO guidance. Thorax. (2018) 73:A94–5. Available online at: https://thorax.bmj.com/content/73/Suppl_4/A94.2

[30] Migliori GB Tiberi S Sotgiu G. Defining the best regimen to treat isoniazid-resistant tuberculosis. Lancet Respir Med. (2018) 6:233–5. 29595499 10.1016/S2213-2600(18)30079-1

[31] Wilson M O'Connor B Matigian N Eather G. Management of isoniazid-monoresistant tuberculosis (Hr-TB) in Queensland, Australia: a retrospective case series. Respir Med. (2020) 173:106163. 33002798 10.1016/j.rmed.2020.106163

[32] Báez-Saldaña R Delgado-Sánchez G García-García L Cruz-Hervert LP Montesinos-Castillo M Ferreyra-Reyes L . Isoniazid mono-resistant tuberculosis: Impact on treatment outcome and survival of pulmonary tuberculosis patients in Southern Mexico 1995–2010. PLoS ONE. (2016) 11:e0168955. 28030600 10.1371/journal.pone.0168955 PMC5193431

[33] Villegas L Otero L Sterling TR Huaman MA Van der Stuyft P Gotuzzo E . Prevalence, risk factors, and treatment outcomes of isoniazid- and rifampicin- mono-resistant pulmonary tuberculosis in Lima, Peru. PLoS ONE. (2016) 11:e0152933. 27045684 10.1371/journal.pone.0152933 PMC4821555

[34] Tabarsi P Baghaei P Hemmati N Mirsaeidi M Kazempour M Mansouri D . Comparison of the effectiveness of 2 treatment regimens in patients with isoniazid-resistant tuberculosis. East Mediterr Health J. (2009) 15:1346–50. 20218123

[35] Escalante P Graviss EA David Musser JM Awe RJ. Treatment of isoniazid-resistant tuberculosis in southeastern Texas. Chest. (2001) 119:1730–6. 10.1378/chest.119.6.1730 11399698

[36] OBabu S Aluoch JA Githui WA Thiong'o R Edwards EA Darbyshire JH . Controlled clinical trial of a regimen of two durations for the treatment of isoniazid resistant pulmonary tuberculosis. Tubercle. (1988) 69:5–14. 3051607 10.1016/0041-3879(88)90035-9

[37] Nolan CM Goldberg SV. Treatment of isoniazid-resistant tuberculosis with isoniazid, rifampin, ethambutol, and pyrazinamide for 6 months. Int J Tuberc Lung Dis. (2002) 6:952–8. 12475140

[38] Binkhamis K Bahatheg MA Altahan FA Alwakeel SS Almutairi KM Alsaeed AA . Prevalence and outcome of isoniazid-monoresistant tuberculosis at a university hospital in Saudi Arabia. Saudi Med J. (2021) 42:636–42. 34078725 10.15537/smj.2021.42.6.20200832 PMC9149726

[39] Van der Heijden YF Karim F G Mufamadi L Zako T Chinappa Shepherd BE . Isoniazid-monoresistant tuberculosis is associated with poor treatment outcomes in Durban, South Africa. Int J Tuberc Lung Dis. (2017) 211:670–6. 28482962 10.5588/ijtld.16.0843 PMC5536436

[40] Hoopes AJ Kammerer JS Harrington TA TM M Ijaz K Armstrong LR. Isoniazid-Monoresistant Tuberculosis in the United States, 1993 to 2003. Arch Intern Med. (2008) 168:1984–92. 18852399 10.1001/archinte.168.18.1984

[41] Munang ML Kariuki M Dedicoat M. Isoniazid-resistant tuberculosis in Birmingham, United Kingdom, 1999–2010. QJM. (2014) 108:19–25. Available online at: https://academic.oup.com/qjmed/article/108/1/19/1588916 24989780 10.1093/qjmed/hcu139

[42] Cattamanchi A Dantes R Metcalfe JZ Jarlsberg LG Grinsdale J Kawamura LM . Clinical characteristics and treatment outcomes of patients with isoniazid-monoresistant tuberculosis. Clin Infect Dis. (2009) 48:179–85. 19086909 10.1086/595689 PMC2756509

[43] Fox L Kramer MR Haim I Priess R Metvachuk A Shitrit D. Comparison of isoniazid monoresistant tuberculosis with drug-susceptible tuberculosis and multidrug-resistant tuberculosis. Euro J Clin Microbiol Infect Dis. (2011) 30:863–7. 21431989 10.1007/s10096-011-1167-4

[44] Reves R Heilig CM Tapy JM Bozeman L Kyle RA Hamilton CD . Intermittent tuberculosis treatment for patients with isoniazid intolerance or drug resistance. Int J Tuberc Lung Dis. (2014) 18:571–80. 24903795 10.5588/ijtld.13.0304

[45] Kim YH Suh GY Chung MP Kim H Kwon OJ Lim SY . Treatment of isoniazid-resistant pulmonary tuberculosis. BMC Infect Dis. (2008) 8:6. 18211720 10.1186/1471-2334-8-6 PMC2245946

[46] Garcia-Prats AJ Du Plessis L Draper H Burger A Seddon JM Zimri K . Outcome of culture-confirmed isoniazid-resistant rifampicin-susceptible tuberculosis in children. Int J Tuberc Lung Dis. (2016) 20:1469–76. 10.5588/ijtld.16.0293 27776587

[47] Nagu TJ Aboud S Matee M Maeurer M Fawzi WW Mugusi F. Effects of isoniazid resistance on TB treatment outcomes under programmatic conditions in a high-TB and -HIV setting: a prospective multicentre study. J Antimicrob Chemother. (2016). 27999054 10.1093/jac/dkw503

[48] Pa L Moser KS. Isoniazid- and rifampin-resistant tuberculosis in San Diego County, California, United States, 1993–2002. Int J Tuberc Lung Dis. (2005) 9:501–6. 15875920

[49] Thai PVK Ha DTM Hanh NT Day J Dunstan S Nhu NTQ . Bacterial risk factors for treatment failure and relapse among patients with isoniazid resistant tuberculosis. BMC Infect Dis. (2018) 18:112. 10.1186/s12879-018-3033-9 29510687 PMC5840777

[50] Huyen MNT Cobelens F Buu TN Lan H Dung ND Kremer K . Epidemiology of isoniazid resistance mutations and their effect on tuberculosis treatment outcomes. Antimicrob Agents Chemother. (2013) 57:3620–7. 23689727 10.1128/AAC.00077-13 PMC3719713

[51] Ormerod LP Horsfield N Green RM. Can a nine-month regimen be used to treat isoniazid resistant tuberculosis diagnosed after standard treatment is started? J Infect. (2001) 42:1–3. 11243745 10.1053/jinf.2000.0773

[52] Bai KJ Yu MY Suo J Chiang CC Chiang IH Lin TH . Short-course chemotherapy for isoniazid-resistant pulmonary tuberculosis. J Formosan Med Assoc. (1998) 97:278–82. 9585680

[53] Salindri AD Sales RMF DiMiceli L Schechter MC Kempker RR Magee MJ. Isoniazid monoresistance and rate of culture conversion among patients in the state of georgia with confirmed tuberculosis, 2009–2014. Ann Am Thoracic Soc. (2018) 15:331–40. 29131662 10.1513/AnnalsATS.201702-147OC PMC5880520

[54] Lai CC Tai CK Huang YT Liao CH Hsueh PR. Isoniazid-resistant tuberculosis, Taiwan, 2000–2010. Emerg Infect Dis. (2011) 17:1769–70. 21888822 10.3201/eid1709.110447 PMC3322092

[55] Maguire H Brailsford S Carless J Yates M Altass L Yates S . Large outbreak of isoniazid-monoresistant tuberculosis in London, 1995 to 2006: case-control study and recommendations. Eurosurveillance. (2011) 16:19830. 21489373

[56] Cornejo Garcia JG Alarcón Guizado VA Mendoza Ticona A Alarcon E Heldal E Moore DAJ. Treatment outcomes for Isoniazid-monoresistant Tuberculosis in Peru, 2012-2014. PLoS ONE. (2018) 13:e0206658. 10.1371/journal.pone.0206658 30513085 PMC6279036

[57] Edwards BD Edwards J Cooper R Kunimoto D Somayaji R Fisher D. Incidence, treatment, and outcomes of Isoniazid mono-resistant Mycobacterium Tuberculosis infections in Alberta, Canada from 2007-2017. PLoS ONE. (2020) 15:e0229691. 10.1371/journal.pone.0229691 32155169 PMC7064215

[58] Kim S Lee H Park HY Jeon K Huh HJ Lee NY . Outcomes of pulmonary Tuberculosis in patients with discordant phenotypic Isoniazid resistance testing. Respir Med. (2017) 133:6–11. 10.1016/j.rmed.2017.11.004 29173450

[59] Lee H Jeong B-H Park HY Jeon K Huh HJ Lee NY . Treatment outcomes with fluoroquinolone-containing regimens for isoniazid-resistant pulmonary tuberculosis. Antimicrobial Agents Chemother. (2016) 60:471–7. 10.1128/AAC.01377-15 26525801 PMC4704191

[60] Romanowski K Chiang LY Roth DZ Krajden M Tang P Cook VJ . Treatment outcomes for Isoniazid-resistant Tuberculosis under program conditions in British Columbia, Canada. BMC Infect Dis. (2017) 17:604. 10.1186/s12879-017-2706-0 28870175 PMC5583994

[61] Bang D Andersen PH Andersen ÅB Thomsen VØ. Isoniazid-resistant Tuberculosis in Denmark: Mutations, transmission and treatment outcome. J Infect. (2010) 60:452–7. 10.1016/j.jinf.2010.03.017 20347869

[62] Saito W Nagayama N Miyamoto M Hara H Suzuki J Masuda K . [Characteristics and treatment outcomes of INH-resistant or RFP-resistant tuberculosis]. Kekkaku(Tuberculosis). (2003) 78:611–7. 14621568

[63] Bachir M Guglielmetti L Tunesi S Billard-Pomares T Chiesi S Jaffré J . Isoniazid-monoresistant tuberculosis in France: Risk factors, treatment outcomes and adverse events. Int J Infect Dis. (2021) 107:86–91. 33823278 10.1016/j.ijid.2021.03.093

[64] Kwak SH Choi JS Lee EH Lee SH Leem AY Lee SH . Characteristics and treatment outcomes of isoniazid mono-resistant tuberculosis: a retrospective study. Yonsei Med J. (2020) 61:1034. 10.3349/ymj.2020.61.12.1034 33251777 PMC7700875

