# Supplementary material for: Effectiveness and safety of Levofloxacin containing regimen in the treatment of Isoniazid mono-resistant pulmonary Tuberculosis: a systematic review
Source: Front Med (Lausanne). 2023 Jun 20;10:1085010. doi: 10.3389/fmed.2023.1085010 (PMC10321706; doi:10.3389/fmed.2023.1085010)
Supplement: Supplementary file 1 [file Table_1.DOCX]

**Supplement.1 Search Strategies**

**Search strategy for PUBMED**

Top of Form

| **Search** | **Query** | **Results** |
| --- | --- | --- |
| #10 | Search: **(((((("Isoniazid resistance tuberculosis"[Title/Abstract]) OR ("Isoniazid resistance pulmonary tuberculosis"[Title/Abstract])) OR ("pulmonary tuberculosis"[Title/Abstract])) OR (((pulmonary tuberculoses[MeSH Terms]) OR (pulmonary tuberculosis[MeSH Terms])) OR (isoniazid[MeSH Terms]))) OR (("pulmonary tuberculoses"[Title/Abstract]) OR (pulmonary tuberculosis[Title/Abstract]))) AND ((((Levofloxacin[Title/Abstract]) OR (isoniazid[Title/Abstract])) OR ("HRZE/RZE"[Title/Abstract])) OR ((levofloxacin[MeSH Terms]) OR (isoniazid[MeSH Terms])))) AND (((((((cure) OR (failure*)) OR (relaps*)) OR (mortality)) OR ("disease progression to MDR")) OR ("additional drug resistance")) OR ("adverse events"))** | [1,937](https://pubmed.ncbi.nlm.nih.gov/?term=%28%28%28%28%28%28%22Isoniazid+resistance+tuberculosis%22%5BTitle%2FAbstract%5D%29+OR+%28%22Isoniazid+resistance+pulmonary+tuberculosis%22%5BTitle%2FAbstract%5D%29%29+OR+%28%22pulmonary+tuberculosis%22%5BTitle%2FAbstract%5D%29%29+OR+%28%28%28pulmonary+tuberculoses%5BMeSH+Terms%5D%29+OR+%28pulmonary+tuberculosis%5BMeSH+Terms%5D%29%29+OR+%28isoniazid%5BMeSH+Terms%5D%29%29%29+OR+%28%28%22pulmonary+tuberculoses%22%5BTitle%2FAbstract%5D%29+OR+%28pulmonary+tuberculosis%5BTitle%2FAbstract%5D%29%29%29+AND+%28%28%28%28Levofloxacin%5BTitle%2FAbstract%5D%29+OR+%28isoniazid%5BTitle%2FAbstract%5D%29%29+OR+%28%22HRZE%2FRZE%22%5BTitle%2FAbstract%5D%29%29+OR+%28%28levofloxacin%5BMeSH+Terms%5D%29+OR+%28isoniazid%5BMeSH+Terms%5D%29%29%29%29+AND+%28%28%28%28%28%28%28cure%29+OR+%28failure%2A%29%29+OR+%28relaps%2A%29%29+OR+%28mortality%29%29+OR+%28%22disease+progression+to+MDR%22%29%29+OR+%28%22additional+drug+resistance%22%29%29+OR+%28%22adverse+events%22%29%29&sort=) |
| #9 | Search: **((((((cure) OR (failure*)) OR (relaps*)) OR (mortality)) OR ("disease progression to MDR")) OR ("additional drug resistance")) OR ("adverse events")** | [2,585,609](https://pubmed.ncbi.nlm.nih.gov/?term=%28%28%28%28%28%28cure%29+OR+%28failure%2A%29%29+OR+%28relaps%2A%29%29+OR+%28mortality%29%29+OR+%28%22disease+progression+to+MDR%22%29%29+OR+%28%22additional+drug+resistance%22%29%29+OR+%28%22adverse+events%22%29&sort=) |
| #8 | Search: **((((("Isoniazid resistance tuberculosis"[Title/Abstract]) OR ("Isoniazid resistance pulmonary tuberculosis"[Title/Abstract])) OR ("pulmonary tuberculosis"[Title/Abstract])) OR (((pulmonary tuberculoses[MeSH Terms]) OR (pulmonary tuberculosis[MeSH Terms])) OR (isoniazid[MeSH Terms]))) OR (("pulmonary tuberculoses"[Title/Abstract]) OR (pulmonary tuberculosis[Title/Abstract]))) AND ((((Levofloxacin[Title/Abstract]) OR (isoniazid[Title/Abstract])) OR ("HRZE/RZE"[Title/Abstract])) OR ((levofloxacin[MeSH Terms]) OR (isoniazid[MeSH Terms])))** | [20,661](https://pubmed.ncbi.nlm.nih.gov/?term=%28%28%28%28%28%22Isoniazid+resistance+tuberculosis%22%5BTitle%2FAbstract%5D%29+OR+%28%22Isoniazid+resistance+pulmonary+tuberculosis%22%5BTitle%2FAbstract%5D%29%29+OR+%28%22pulmonary+tuberculosis%22%5BTitle%2FAbstract%5D%29%29+OR+%28%28%28pulmonary+tuberculoses%5BMeSH+Terms%5D%29+OR+%28pulmonary+tuberculosis%5BMeSH+Terms%5D%29%29+OR+%28isoniazid%5BMeSH+Terms%5D%29%29%29+OR+%28%28%22pulmonary+tuberculoses%22%5BTitle%2FAbstract%5D%29+OR+%28pulmonary+tuberculosis%5BTitle%2FAbstract%5D%29%29%29+AND+%28%28%28%28Levofloxacin%5BTitle%2FAbstract%5D%29+OR+%28isoniazid%5BTitle%2FAbstract%5D%29%29+OR+%28%22HRZE%2FRZE%22%5BTitle%2FAbstract%5D%29%29+OR+%28%28levofloxacin%5BMeSH+Terms%5D%29+OR+%28isoniazid%5BMeSH+Terms%5D%29%29%29&sort=) |
| #7 | Search: **(((Levofloxacin[Title/Abstract]) OR (isoniazid[Title/Abstract])) OR ("HRZE/RZE"[Title/Abstract])) OR ((levofloxacin[MeSH Terms]) OR (isoniazid[MeSH Terms]))** | [34,381](https://pubmed.ncbi.nlm.nih.gov/?term=%28%28%28Levofloxacin%5BTitle%2FAbstract%5D%29+OR+%28isoniazid%5BTitle%2FAbstract%5D%29%29+OR+%28%22HRZE%2FRZE%22%5BTitle%2FAbstract%5D%29%29+OR+%28%28levofloxacin%5BMeSH+Terms%5D%29+OR+%28isoniazid%5BMeSH+Terms%5D%29%29&sort=) |
| #6 | Search: **(((("Isoniazid resistance tuberculosis"[Title/Abstract]) OR ("Isoniazid resistance pulmonary tuberculosis"[Title/Abstract])) OR ("pulmonary tuberculosis"[Title/Abstract])) OR (((pulmonary tuberculoses[MeSH Terms]) OR (pulmonary tuberculosis[MeSH Terms])) OR (isoniazid[MeSH Terms]))) OR (("pulmonary tuberculoses"[Title/Abstract]) OR (pulmonary tuberculosis[Title/Abstract]))** | [97,212](https://pubmed.ncbi.nlm.nih.gov/?term=%28%28%28%28%22Isoniazid+resistance+tuberculosis%22%5BTitle%2FAbstract%5D%29+OR+%28%22Isoniazid+resistance+pulmonary+tuberculosis%22%5BTitle%2FAbstract%5D%29%29+OR+%28%22pulmonary+tuberculosis%22%5BTitle%2FAbstract%5D%29%29+OR+%28%28%28pulmonary+tuberculoses%5BMeSH+Terms%5D%29+OR+%28pulmonary+tuberculosis%5BMeSH+Terms%5D%29%29+OR+%28isoniazid%5BMeSH+Terms%5D%29%29%29+OR+%28%28%22pulmonary+tuberculoses%22%5BTitle%2FAbstract%5D%29+OR+%28pulmonary+tuberculosis%5BTitle%2FAbstract%5D%29%29&sort=) |
| #5 | Search: **(levofloxacin[MeSH Terms]) OR (isoniazid[MeSH Terms])** | [22,801](https://pubmed.ncbi.nlm.nih.gov/?term=%28levofloxacin%5BMeSH+Terms%5D%29+OR+%28isoniazid%5BMeSH+Terms%5D%29&sort=) |
| #4 | Search: **((Levofloxacin[Title/Abstract]) OR (isoniazid[Title/Abstract])) OR ("HRZE/RZE"[Title/Abstract])** | [26,656](https://pubmed.ncbi.nlm.nih.gov/?term=%28%28Levofloxacin%5BTitle%2FAbstract%5D%29+OR+%28isoniazid%5BTitle%2FAbstract%5D%29%29+OR+%28%22HRZE%2FRZE%22%5BTitle%2FAbstract%5D%29&sort=) |
| #3 | Search: **("pulmonary tuberculoses"[Title/Abstract]) OR (pulmonary tuberculosis[Title/Abstract])** | [34,741](https://pubmed.ncbi.nlm.nih.gov/?term=%28%22pulmonary+tuberculoses%22%5BTitle%2FAbstract%5D%29+OR+%28pulmonary+tuberculosis%5BTitle%2FAbstract%5D%29&sort=) |
| #2 | Search: **((pulmonary tuberculoses[MeSH Terms]) OR (pulmonary tuberculosis[MeSH Terms])) OR (isoniazid[MeSH Terms])** | [90,439](https://pubmed.ncbi.nlm.nih.gov/?term=%28%28pulmonary+tuberculoses%5BMeSH+Terms%5D%29+OR+%28pulmonary+tuberculosis%5BMeSH+Terms%5D%29%29+OR+%28isoniazid%5BMeSH+Terms%5D%29&sort=) |
| #1 | Search: **(("Isoniazid resistance tuberculosis"[Title/Abstract]) OR ("Isoniazid resistance pulmonary tuberculosis"[Title/Abstract])) OR ("pulmonary tuberculosis"[Title/Abstract])** | [34,728](https://pubmed.ncbi.nlm.nih.gov/?term=%28%28%22Isoniazid+resistance+tuberculosis%22%5BTitle%2FAbstract%5D%29+OR+%28%22Isoniazid+resistance+pulmonary+tuberculosis%22%5BTitle%2FAbstract%5D%29%29+OR+%28%22pulmonary+tuberculosis%22%5BTitle%2FAbstract%5D%29&sort=) |

**Search strategy for Embase**

| 12. | #1 AND #2 AND #3 AND #4 AND #5 AND #6 AND #7 AND #8 AND #9  AND #10 AND #11 | [2,585](https://www.embase.com/) |
| --- | --- | --- |
| 11. | **'efficacy'**/exp OR **efficacy** OR **effectiveness** OR **progress** OR **worse** OR **'outcome'**/exp OR **outcome** OR **'success'**/exp OR **success** OR **improve** | [6,678,776](https://www.embase.com/) |
| 10.  9.  8.  7.  6.  5.  4.  3.  2.  1. | #8 AND #9  ATT OR (**anti** AND (**'tuberculosis'**/exp OR **tuberculosis**)) OR **'anti tuberculosis'** OR **'dots'**/exp OR **dots** OR **'directly observed treatment'** OR (**directly** AND **observed** AND (**'treatment'**/exp OR **treatment**)) OR (**category** AND **1**) OR **'category 1'** OR **'category one'** OR **'rifampicin'**/exp OR **rifampicin** OR **'ethambutol'**/exp OR **ethambutol** OR **'pyrazinamide'**/exp OR **pyrazinamide** OR **'tuberculous drug therapy'** OR (**tuberculous** AND (**'drug'**/exp OR **drug**) AND (**'therapy'**/exp OR **therapy**)) OR **'intermittent therapy'** OR (**intermittent** AND (**'therapy'**/exp OR **therapy**)) OR **'intermittent att'** OR (**intermittent** AND **att**) OR **'intermittent tb drug'** OR (**intermittent** AND (**'tb'**/exp OR **tb**) AND (**'drug'**/exp OR **drug**)) OR **'intermittent tuberculosis'** OR (**intermittent** AND (**'tuberculosis'**/exp OR **tuberculosis**)) OR **'first line att'** OR (**first** AND (**'line'**/exp OR **line**) AND **att**) OR **'first line tb treatment'** OR (**first** AND (**'line'**/exp OR **line**) AND (**'tb'**/exp OR **tb**) AND (**'treatment'**/exp OR **treatment**)) OR **firstline** OR **'antitubercular agents'**/exp OR **'antitubercular agents'** OR (**antitubercular** AND **agents**) OR **'therapeutic use'**/exp OR **'therapeutic use'** OR (**therapeutic** AND **use**)  [976,585](https://www.embase.com/)  #6 AND #7 [7,070](https://www.embase.com/)  **'fluoroquinolones'**/exp OR **fluoroquinolones** OR **'levofloxacin'**/exp OR **levofloxacin** OR **'quinolones'**/exp OR **quinolones** OR **'therapeutic use'**/exp OR **'therapeutic use'** OR (**therapeutic** AND **use**)  [563,503](https://www.embase.com/)  #4 AND #5 [44,910](https://www.embase.com/)  **'tuberculosis multidrug-resistant'**/exp OR **'tuberculosis multidrug-resistant'** OR ((**'tuberculosis'**/exp OR **tuberculosis**) AND **'multidrug resistant'**) OR **'drug therapy'**/exp OR **'drug therapy'** OR ((**'drug'**/exp OR **drug**) AND (**'therapy'**/exp OR **therapy**)) OR **'tuberculosis pulmonary'**/exp OR **'tuberculosis pulmonary'** OR ((**'tuberculosis'**/exp OR **tuberculosis**) AND **pulmonary**)  [7,435,174](https://www.embase.com/)  #1 AND #2 AND #3  [71,431](https://www.embase.com/)  **'inh tuberculosis'** OR ((**'inh'**/exp OR **inh**) AND (**'tuberculosis'**/exp OR **tuberculosis**)) OR **'isoniazid'**/exp OR **isoniazid** OR **'drug resistant'**  [127,911](https://www.embase.com/)  **'inh'**/exp OR **inh** OR **'isoniazid'**/exp OR **isoniazid** OR **'drug resistant'**  [132,134](https://www.embase.com/)  **Other possible terms for population**  **'drug resistant tuberculosis'**/exp OR **'drug resistant tuberculosis'** OR ((**'drug'**/exp OR **drug**) AND **resistant** AND (**'tuberculosis'**/exp OR **tuberculosis**)) OR ((**'drug'**/exp OR **drug**) AND **resistant**) OR **'drug resistant'** OR **'poly resistant tuberculosis'** OR (**poly** AND **resistant** AND (**'tuberculosis'**/exp OR **tuberculosis**)) OR (**poly** AND **resistant**) OR **'poly resistant'** OR **polyresistant** OR **'mono resistant tuberculosis'** OR (**mono** AND **resistant** AND (**'tuberculosis'**/exp OR **tuberculosis**)) OR (**mono** AND **resistant**) OR **'mono resistant'** OR **monoresistan** OR **'hr tb'** OR **'inh mono'** OR ((**'inh'**/exp OR **inh**) AND **mono**) OR **'h mono'** OR (**h** AND **mono**) OR **hrtb**  [438,234](https://www.embase.com/) | 5143 |

**Search strategy for Cochrane Library**

601 Trials matching "pulmonary tuberculoses" OR "pulmonary tuberculosis" OR "Isoniazid resistance tuberculosis" OR "Isoniazid resistance pulmonary tuberculosis" in Title Abstract Keyword AND "levofloxacin" OR "Ofloxacin, (S)-Isomer" OR "Levofloxacin-containing product" OR "(-)-Ofloxacin" OR "(S)-9-Fluoro-2,3-dihydro-3-methyl-10-(4-methyl-1-piperazinyl)-7-oxo-7H-pyrido(1,2,3-de)-1,4-benzoxazine-6-carboxylic acid" OR "(S)-Ofloxacin" OR "Ofloxacin S-(-)-form" OR "(3S)-(-)-9-fluoro-3-methyl-10-(4-methyl-1-piperazinyl)-7-oxo-2,3-dihydro-7H-pyrido[1,2,3-de][1,4]benzoxazine-6-carboxylic acid" OR "(S)-(-)-9-fluoro-3-methyl-10-(4-methyl-1-piperazinyl)-7-oxo-2,3-dihydro-7H-pyrido[1,2,3-de][1,4]benzooxazine-6-carboxylic acid" OR "L-Ofloxacin" OR "Levofloxacine" OR "Levofloxacino" OR "Levofloxacinum" OR "(S)-9-Fluoro-2,3-dihydro-3-methyl-10-(4-methyl-1-piperazinyl)-7-oxo-7H-pyrido(1,2,3-de)-1,4-benzoxazine-6-carboxylic Acid Hydrate (2:1)" OR "Levofloxacin Hydrate" OR "7H-Pyrido(1,2,3-de)-1,4-benzoxazine-6-carboxylic Acid, 9-Fluoro-2,3-dihydro-3-methyl-10-(4-methyl-1-piperazinyl)-7-oxo-, Hydrate (2:1), (S)-" OR "isoniazid" OR "HRZE/RZE" OR "DOTS therapy" in Title Abstract Keyword - (Word variations have been searched
