# Supplementary material for: Effectiveness and safety of Levofloxacin containing regimen in the treatment of Isoniazid mono-resistant pulmonary Tuberculosis: a systematic review
Source: Front Med (Lausanne). 2023 Jun 20;10:1085010. doi: 10.3389/fmed.2023.1085010 (PMC10321706; doi:10.3389/fmed.2023.1085010)
Supplement: Supplementary file 2 [file Table_2.DOCX]

**Supplement. 2 Data extraction sheet**

1. Study ID :
2. Author & Year of publication (Surname with initials a& year) :
3. Title:
4. Year/month of study (from enrollment to outcome assessment)
5. Country
6. Setting
   - Outpatient/ In-patient:
   - Tertiary/ district/ secondary/tertiary hospital:
   - Clinical/Programmatic :
   - Clinical/ Public/ Private/NGO:
   - Description of the setting :
   - TB prevalence in the setting :
   - Prev. INH mono resistance in the setting :

7. Design & Population

- Design (RCT/ non RCT/Cohort – retro/pros-retro/ with or without control arm):
- Patient age group (<15/>15/ both) :
- Sex group (Males/Females/ Third genders/All):
- Mean/ Median (Sd/IQR) age:
- age distribution if mentioned (age group wise percentage) :
- ATT (daily/ intermittent)
- HIV status (Positive / Negative/unknown)
- DM status (Positive /Negative / unknown)
- Tb type (New/ retreatment) or both
- follow up after the treatment (Yes/No):
- Months of follow up (in months)

1. Intervention

- Levofloxacin with first line ATT arm ( Yes/No)
- Control group has only first line ATT ( Yes/No)

*No. in intervention (Lfx) group :*

- No. with declared cure :
- No. treatment completed :
- No. failure during treatment or follow up:
- No. relapse during treatment or follow up
- No. of deaths
- No. of defaulters
- No. acquired Lfx resistance :
- No. toxicity reported due to Lfx

*No. in the control group:*

- No. with declared cure:
- No. treatment completed:
- No. failure during treatment or follow up:
- No. relapse during treatment or follow up
- No. of deaths
- No. of defaulters
- No. toxicity reported with ATT:

9. Statistical analysis outcomes :

Association between intervention and control :

Association described between two arms ( Yes/No) :

b. Association between two arms (Outcome – cure) :

- If available OR or RR/aOR or aRR :
- SE or CI:
- IF adjusted, no. of confounders adjusted for:
- Mention the confounders adjusted:

b. Association between two arms (Outcome – treatment completed) :

- If available OR or RR/aOR or aRR
- SE or CI :
- IF adjusted, no. of confounders adjusted for
- Mention the confounders adjusted

c. Association between two arms (Outcome – treatment failure ) :

- If available OR or RR/aOR or aRR
- SE or CI :
- IF adjusted, no. of confounders adjusted for
- Mention the confounders adjusted

d. Association between two arms (Outcome – relapse ) :

- If available OR or RR/aOR or aRR
- SE or CI :
- IF adjusted, no. of confounders adjusted for
- Mention the confounders adjusted

e. Association between two arms (Outcome – death ) :

- If available OR or RR/aOR or aRR :
- SE or CI :
- IF adjusted, no. of confounders adjusted for
- Mention the confounders adjusted

f. Association between two arms (Outcome – default) :

- If available OR or RR/aOR or aRR
- SE or CI :
- IF adjusted, no. of confounders adjusted for
- Mention the confounders adjusted

h. Association between two arms (Outcome – progression to MDR) :

- If available OR or RR/aOR or aRR
- SE or CI :
- IF adjusted, no. of confounders adjusted for
- Mention the confounders adjusted

i. Association between two arms (Outcome – toxicity ) :

- If available OR or RR/aOR or aRR
- SE or CI :
- IF adjusted, no. of confounders adjusted for
- Mention the confounders adjusted

10.Risk of bias for cohort study

1. Representativeness of exposed cohort (truly representative/somewhat representative/selected group of users/not representative/no description of derviation of cohort) :
2. Selection of unexposed cohort (drawn from same community as exposed/drawn from a different source/no description) :
3. Exposure ascertainment (secure record or measurement / structured interview / written self report/ no description) :
4. Demonstrated that outcome of interest was not present at start of study (yes/no/not clear) :
5. Adjustment of confounders (age / sex / TB site / TB microbilogical status / new or old TB / baseline BMI / baseline anemia / HIV status / Baseline tobacco / baseline alcohol) If yes, how many and which one?
6. Comparability of cohort / confounders Adjustement for other confounders ? mention which one?
7. Adjustment for at least four imprtant confounders (age / sex / TB site / TB microbilogical status / new or old TB / baseline BMI / baseline anemia / HIV status / Baseline tobacco / baseline alcohol) Yes / no / not clear) :
8. outcome assessment (independent blind / record review or linkage / self report / no description) :
9. Adequacy of follow up (complete - all subjects accounted for / no statement :

11.Risk of bias for interventional studies :

1. sequence generation (bias: low, unclear, high)
2. allocation concealment (bias: low, unclear, high)
3. blinding of participants (bias: low, unclear, high)
4. personnel and outcome assessors (bias: low, unclear, high)
5. incomplete outcome data (bias: low, unclear, high)
6. selective outcome reporting (bias: low, unclear, high)
7. other sources of bias. (bias: low, unclear, high)

12. Miscellaneous

- Does the study fit into RQ? :
- Conflict of interest statement
- Ethics statement mentioned and is clear
- Additional comments
- corresponding author email and other contact details esp phone number. Any other author contact details
- Need to write to him/her?
- If yes for what? Date when email sent
- Next follow up when and by whom, details :
